# *Clostridium butyricum* MIYAIRI 588 Increases the Lifespan and Multiple-Stress Resistance of *Caenorhabditis elegans*

**DOI:** 10.3390/nu10121921

**Published:** 2018-12-05

**Authors:** Maiko Kato, Yumi Hamazaki, Simo Sun, Yoshikazu Nishikawa, Eriko Kage-Nakadai

**Affiliations:** Graduate School of Human Life Science, Osaka City University, Osaka 558-8585, Japan; chisukonya@yahoo.co.jp (M.K.); yumi.hamazaki123@gmail.com (Y.H.); sunsimo1986511@gmail.com (S.S.)

**Keywords:** *Clostridium butyricum*, lifespan, stress resistance, *Caenorhabditis elegans*

## Abstract

*Clostridium butyricum* MIYAIRI 588 (CBM 588), one of the probiotic bacterial strains used for humans and domestic animals, has been reported to exert a variety of beneficial health effects. The effect of this probiotic on lifespan, however, is unknown. In the present study, we investigated the effect of CBM 588 on lifespan and multiple-stress resistance using *Caenorhabditis elegans* as a model animal. When adult *C. elegans* were fed a standard diet of *Escherichia coli* OP50 or CBM 588, the lifespan of the animals fed CBM 588 was significantly longer than that of animals fed OP50. In addition, the animals fed CBM588 exhibited higher locomotion at every age tested. Moreover, the worms fed CBM 588 were more resistant to certain stressors, including infections with pathogenic bacteria, UV irradiation, and the metal stressor Cu^2+^. CBM 588 failed to extend the lifespan of the *daf-2*/insulin-like receptor, *daf-16*/FOXO and *skn-1*/Nrf2 mutants. In conclusion, CBM 588 extends the lifespan of *C. elegans* probably through regulation of the insulin/IGF-1 signaling (IIS) pathway and the Nrf2 transcription factor, and CBM 588 improves resistance to several stressors in *C. elegans*.

## 1. Introduction

Probiotics are defined as live microorganisms that, when administered in adequate amounts, confer health benefits to the host [1]. *Clostridium butyricum*, which is an anaerobic, Gram-positive, spore-forming bacillus, is one of the commercialized probiotic bacteria. *C. butyricum* is distributed in a variety of environments and in the gut of various animals, including humans [2]. Several studies have described the prevalence of *C. butyricum*; 44% of fecal samples collected from asymptomatic preterm neonates were positive for *C. butyricum* [3], and *C. butyricum* was identified in 10% to 20% of human fecal samples by microbial culture [4].

*C. butyricum* MIYAIRI 588 (CBM 588) is used as an anti-diarrheal medicine in Japan. In clinical practice, the administration of CBM 588 was shown to prevent antibiotic-associated diarrhea in children [5]. Numerous experimental studies have demonstrated the beneficial properties of this probiotic, including the induction of interleukin-10 (IL-10)-producing macrophages in an experimental mouse model of colitis [6], the promotion of regulatory T-cell generation in the intestine through transformation of growth factor-ß (TGF-ß) from dendritic cells [7], the improvement of high-fat diet-induced non-alcoholic fatty liver disease in rats [8], the inhibition of the cytotoxic effect of *Clostridium difficile* in vitro [9], and the prevention of enterohemorrhagic *Escherichia coli* O157:H7 infection [10] and gastric ulcers in mice [11]. The effect of *C. butyricum* on longevity, however, is unknown.

*Caenorhabditis elegans*, a free-living nematode that feeds on bacteria, is a powerful experimental model due to its ease of cultivation, its short and reproducible lifespan, and the abundance of genetic tools available for studying this organism [12]. Although *C. elegans* was originally isolated from rich soil or compost [13], where it is mostly found in the dauer stage [14,15], feeding and reproducing stages of this species have been found in microbe-rich habitats such as rotting fruit and plant matter [16,17]. Three independent studies that provided the first description of the microbiome of *C. elegans* [18,19,20], and a meta-analysis of the data [21], showing possible functions of the naturally associated bacteria; e.g., the microbes enhance *C. elegans* fitness under standard and also stressful conditions, and confer resistance to infection [19]. Specific microbes correlate with the population state of *C. elegans* and the bacterial influence is not simply nutritional [20]. In the laboratory, *C. elegans* is typically maintained on agar plates and fed *Escherichia coli* laboratory strains (e.g., OP50) [22]. We previously showed that feeding on lactic acid bacteria, including lactobacilli and bifidobacteria, extends the lifespan of worms compared with feeding on *E. coli* OP50 [23]. Afterwards, several lines of investigation addressed the interactions between worms and probiotic bacteria, yielding insights into mechanisms by which probiotic bacteria enhance immune responses and increase lifespan in the *C. elegans* host [24,25]. Thus, *C. elegans* has been an accepted model for investigating dietary manipulation with probiotics [26,27].

In the present study, we examined the influence of CBM 588 on the lifespan of *C. elegans*. CBM 588 was shown to extend the lifespan probably through regulation of the insulin/IGF-1 signaling (IIS) pathway and the Nrf2 transcription factor and to improve resistance to several stressors in *C. elegans*.

## 2. Materials and Methods

### 2.1. Bacterial Strain and Culture Conditions

*Escherichia coli* OP50, which is used as the standard feed for *C. elegans* cultivation, was grown on tryptone soya agar (Nissui Pharmaceutical, Tokyo, Japan) at 37 °C. *Clostridium butyricum* MIYAIRI 588 (CBM 588), which was kindly provided by Miyarisan Pharmaceutical, was cultured on GAM agar (Nissui Pharmaceutical, Japan). The *Salmonella enterica* serovar Enteritidis strain SE1, originally isolated from a diarrheal specimen and used as a pathogen, was grown on tryptone soya agar at 37 °C. Similarly, *Staphylococcus aureus* NBRC13276 was cultured on tryptone soya agar. The cultured bacteria were scraped and weighed. Aliquots (100 mg wet weight) of bacteria were suspended in 0.5 mL of M9 buffer (5 mM potassium phosphate, 1 mM CaCl_2_, and 1 mM MgSO_4_). 50 µL of the bacterial suspension was spread onto NGM modified to be peptone free (mNGM) plates (1.7% (*w*/*v*) agar, 50 mM NaCl, 1 mM CaCl_2_, 5 µg/mL cholesterol, 25 mM KH_2_PO_4_, and 1 mM MgSO_4_) (10 mg bacteria per plate), and used in the experimental assays.

### 2.2. C. elegans Strains and Culture Conditions

The wild-type *C. elegans* strain Bristol N2 and the following derivative mutant strains were obtained from the Caenorhabditis Genetics Center: CB1370 *daf-2*(*e1370*), no outcross; CF1038 *daf-16(mu86)*, outcrossed with N2 eleven times by the Kenyon lab.; and VC1772 *skn-1*(*ok2315*), outcrossed one time by the Moerman lab. These *C. elegans* strains were maintained using standard techniques [22]. The animals were cultured as follows: eggs were prepared from adult *C. elegans* by exposure to a sodium hypochlorite/sodium hydroxide solution. The egg suspension was incubated in M9 buffer for one day at 25 °C to allow for hatching and synchronization, and the resulting suspension of synchronized L1-stage worms was centrifuged at 156× *g* for 1 min. After the removal of the supernatant by aspiration, the remaining larvae were transferred onto mNGM plates covered with 10 mg of OP50. The transferred worms were cultured at 25 °C for two days (referred to as three-day-old animals). *daf-2*(*e1370*) animals were grown at 15 °C, the permissive temperature, until they reached the young adult stage.

### 2.3. Determination of the C. elegans Lifespan

Lifespan assays were performed as follows: synchronized three-day-old (young adult) animals (35 animals per plate) were placed on a 5-cm mNGM plate covered with 10 mg of OP50 alone, CBM 588 alone, or a mixture of 5 mg each of OP50 and CBM 588, and then, the plates were incubated at 25 °C. The animals were transferred daily to fresh plates for the first four days, and thereafter, they were transferred every other day. The number of live and dead animals was recorded every day. An animal was considered dead when it failed to respond to a gentle touch with a worm picker. Animals that crawled off the plate or died from internal hatching were considered lost and not included in the analysis. Each assay was carried out in duplicate (two plates). Two independent replicates were performed except for experiments using UV-killed bacteria and mutant analysis using *daf-2*(*e1370*). The data from the replicates were merged, and presented. Worm survival was calculated by the Kaplan-Meier method, and differences in survival were analyzed using the log-rank test.

The mean lifespan (MLS) was estimated using the following formula [28]:

(1) $$\mathrm{MLS}=\frac{1}{N}\sum_{j}\frac{x_{j}+x_{j+1}}{2}d_{j}$$

where *d_j_* is the number of worms that died in the age interval (*x_j_* to *x_j_*_+1_), and *N* is the total number of worms. The standard error (SE) of the estimated mean lifespan was calculated using the following equation:

(2) $$\mathrm{SE}=\sqrt{\frac{1}{N(N-1)}\sum_{j}{\left(\frac{x_{j}+x_{j+1}}{2}-\mathrm{MLS}\right)}^{2}d_{j}}$$

Maximum lifespan was calculated as the mean lifespan of the longest-living 15% of the worms in each group.

### 2.4. Measurement of Body Size

Three-day-old adult worms were placed on mNGM plates covered with lawns of OP50, CBM 588, or a mixture of the two strains. The plates were incubated at 25 °C, and the body size of the live worms was measured every 24 h until the worms reached an age of 10 days. Images of adult nematodes were taken with an M165FC stereoscopic microscope (Leica microsystems, Wetzlar, Germany) equipped with a DFC425C camera (Leica microsystems, Germany) and analyzed using ImageJ software. The area of a worm’s projection was used as an index of the body size.

### 2.5. Measurement of Brood Size

Two L4-stage hermaphrodites were transferred to an mNGM plate covered with a lawn of OP50, CBM 588, or a mixture of the two strains. The parental animals were transferred to fresh mNGM plates every 24 h until the end of the reproductive period. The resulting eggs were left to hatch, and the number of progeny was then determined. Two independent replicates were tested to verify reproducibility, and the merged data were statistically analyzed and presented.

### 2.6. Measurement of Locomotion

Three-day-old adult worms were placed on mNGM plates covered with lawns of OP50, CBM 588, or a mixture of the two strains. The plates were incubated at 25 °C, and the body size of live worms was measured every other day from days 7 to 23. The motility of the worms was then examined using the scoring method described in previous reports [25,29,30]. Briefly, worms were classified as class “A” when they exhibited spontaneous movement or vigorous locomotion in response to prodding; class ‘‘B’’ worms were those that did not move unless prodded or appeared to have uncoordinated movement; and class ‘‘C’’ worms were those that moved only their head and/or tail in response to prodding. Dead worms were classified as class “D”. Two independent replicates were tested, and the merged data were statistically analyzed and presented.

### 2.7. Stress Resistance Assays

Worms were grown from an age of 3 days on mNGM plates containing OP50 or CBM 588 and then subjected to stress resistance assays. The bacterial infection was performed as previously shown [23]. Briefly, 7-day-old worms were transferred onto mNGM plate covered with 10 mg of *Staphylococcus aureus* or *Salmonella* instead of OP50 or CBM 588, cultured at 25 °C, and the lifespan was determined. To assess thermal tolerance, 7-day-old worms were incubated at 35 °C for 6 h and then scored for viability every hour. The following assays were performed as previously described [31]. 9-day-old worms were exposed to UV irradiation at 500 J/m^2^ using a HL-2000 HybriLinker (UVP, Upland, CA, USA), incubated at 25 °C, and then scored for viability every day. For the oxidative stress assay, 9-day-old worms were transferred into M9 buffer containing 0.1% cholesterol (5 mg/mL in ethanol) and 2 mM hydrogen peroxide, and then scored for viability every hour. Similarly, for the heavy metal stress assay, 9-day-old worms were transferred to a 7-mM CuCl_2_ solution. Survival was determined by touch-provoked movement. Worms were scored as dead when they failed to respond to repeated touching with a worm picker. The assays were performed at least twice.

### 2.8. RNA Sequencing

Three-day-old worms were cultured for five days on mNGM plates covered with OP50 (control-fed group) or CBM 588 (CBM 588-fed group). Approximately 200 worms in each group were collected and soaked in RNA*later* solution (Qiagen, Hilden, Germany). Total RNA was isolated using the RNeasy Lipid Tissue kit (Qiagen). RNA sequencing was performed by DNAFORM (Yokohama, Japan) as follows: mRNAs were purified using the Magnosphere™ UltraPure mRNA Purification Kit (Clontech, Mountain View, CA, USA). Then, libraries prepared using the SMARTer^®^ Stranded Total RNA-Seq Kit (Clontech) were run on HiSeq as 150 bp paired-end (Illumina Inc., San Diego, CA, USA). The quality of the RNA-seq results was first assessed using FastQC (ver. 0.11.7). The raw reads were trimmed and quality-filtered using the Trim Galore! (ver. 0.4.4), Trimmomatic (ver. 0.36) [32] and cutadapt (ver. 1.16) software. Clean reads were aligned versus the N2 *Caenorhabditis elegans* reference genome ce11 (WBcel235.91) using STAR (ver. 2.6.1a) [33]. Mapping statistics was presented in Table S1. After the read count of gene features was performed with the featureCounts tool (ver. 1.6.1) [34], a quantitative differential expression analysis between conditions was performed using DESeq (ver. 1.30.0) [35] to compare the control-fed and CBM 588-fed groups (see Table S2). Genes with |Log_2_FC| ≥ 2 and *p*-value < 0.05 (Tables S3 and S4) were subjected to the enrichment analysis based on the Gene Ontology (GO) category for Biological Process (BP), Molecular Function (MF), and Cellular Component (CC) using clusterProfiler (ver. 3.6.0) [36] (Tables S5 and S6). The raw and processed data files were deposited at Gene Expression Omnibus (GEO) (GSE123163).

### 2.9. Reverse Transcription and Real-Time PCR

Genomic DNA was removed and cDNA was synthesized using the QuantiTect Reverse Transcription Kit (Qiagen). Real-time PCR (quantitative PCR) was performed with a StepOnePlus Real-Time PCR system (Thermo Fisher Scientific, Waltham, MA, USA) using FastStart Universal SYBR Green Master (ROX) (Roche Diagnostics, Mannheim, Germany) with the following parameters: 95 °C for 10 min, and 40 cycles of 95 °C for 15 s and 60 °C for 1 min. Samples from three biological replicates were analyzed. Relative mRNA was determined with the ∆∆Ct method [37] using expression of the *cyc-1* or *tba-1* gene. The primers used for real-time PCR were as follows: *cyc-1*, (F: Forward) 5’-CGTGGTTCAAGGATCTAAACG-3’ and (R: Reverse) 5’-ACCGAGTTCTCCAAAGCGTA-3’; *tba-1*, (F) 5’-TCAACACTGCCATCGCCGCC-3’ and (R) 5’-TCCAAGCGAGACCAGGCTTCAG-3’; *gsto-1*, (F) 5’-AGCTGCTAGCGGACAGGTTA-3’ and (R) 5’-CGCTGCTTTTCATCTTTCAA-3’; and *gsto-2*, (F) 5’-TTGAACTTGCAAAAGCATATGATAC-3’ and (R) 5’-TGAGATAATCGACAAATCCTGGT-3’; *dod-22*, (F) 5’-CTTCTTCGTCAAACCGCAAT-3’ and (R) 5’-TGTGAGAGACTTCCGATGTAGG-3’; *dod-23*, (F) 5’-GATTCAGTGCGCTGTACCATAC-3’ and (R) 5’-GTAGGCTTCCATCTTTGAGAGC-3’; *dod-24*, (F) 5’-CTTCTTCGTCAAACCGCAAT-3’ and (R) 5’-TGTGAGAGACTTCCGATGTAGG-3’.

## 3. Results and Discussion

### 3.1. CBM 588 Prolonged the Longevity of C. elegans

**Figure 1:**
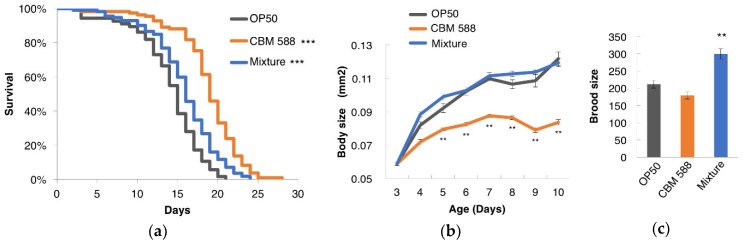
*Clostridium butyricum* MIYAIRI 588 (CBM 588) extended the lifespan of *C. elegans*. Survival curves (**a**), growth curves (**b**) and brood size (**c**) of animals fed OP50 (control), CBM 588, or a mixture of OP50 and CBM 588. (**a**) Worms were 3 days old on the nominal Day 0. OP50 (control)-fed group, *n* = 122; CBM 588-fed group, *n* = 109; mixture-fed group, *n* = 112. Differences in survival were analyzed using the log-rank test. *** *p* < 0.001 vs. control-fed group. Detailed lifespan data and statistics are provided in Table S7. (**b**) The area of the worm’s projection was measured. *n* ≥ 8 for each assay. ** *p* < 0.01 vs. control-fed group, ANOVA followed by post hoc Tukey-Kramer’s test. (**c**) The brood size of worms fed CBM 588 alone was not significantly different in comparison with the size of those fed OP50 alone, and the brood size of the mixture-fed group was larger compared with the control-fed group. OP50 (control), *n* = 11; CBM 588, *n* = 12; mixture, *n* = 13. ** *p* < 0.01 vs. control-fed group, Statistical analysis was performed by ANOVA followed by post hoc Tukey-Kramer’s test.

To examine the effect of *C. butyricum* MIYAIRI 588 (CBM 588) on the *C. elegans* lifespan, worms were fed CBM 588 from an age of 3 days. As a result, the lifespan of the animals fed CBM 588 was significantly longer than that of the animals fed *E. coli* OP50 (Figure 1a). It is possible that the longevity that resulted from the feeding of CBM 588 may be due to dietary restriction (DR), which could be considerable if CBM 588 contains fewer calories or is too difficult for worms to digest. To test this possibility, CBM 588 was mixed with an equal volume of OP50 that was sufficient to avoid DR, and then, the animals were fed this mixture. This mixture of CBM 588 and OP50 also extended the lifespan compared with *E. coli* OP50 alone (Figure 1a). DR is known to decrease body weight and fertility in *C. elegans* [38,39]. The body size of the animals fed CBM 588 alone, but not the mixture of CBM 588 and OP50, was smaller compared with that of animals fed OP50 alone (Figure 1b). The brood size of the worms fed CBM 588 alone was not significantly different in comparison with the size of those fed OP50 alone (Figure 1c). In contrast, the brood size of the mixture-fed group was larger compared with the control-fed group (Figure 1c). Taken together, these findings indicate that CBM 588 prolonged the longevity of *C. elegans* at least partially in a DR-independent manner.

### 3.2. UV-Killed CBM 588 Extended the Lifespan of C. elegans

**Figure 2:**
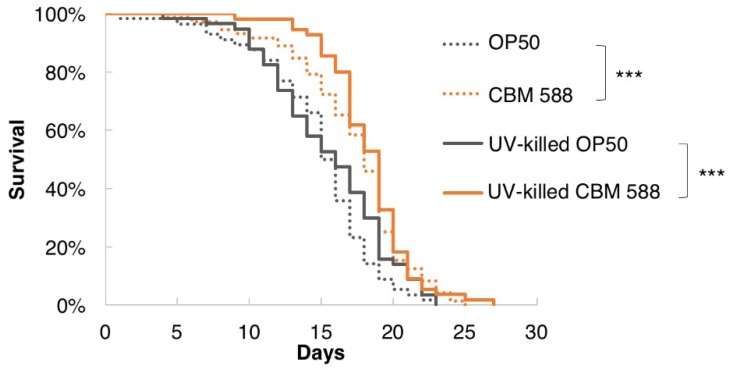
UV-killed CBM 588 prolonged the longevity of *C. elegans.* Survival curves of animals fed UV-killed bacteria. The lifespan of worms fed UV-killed CBM 588 was significantly increased compared with those fed UV-killed OP50. OP50, *n* = 56; CBM 588, *n* = 72; UV-killed OP50, *n* = 57; UV-killed CBM 588, *n* = 55. *** *p* < 0.001, log-rank test. Detailed lifespan data and statistics are provided in Table S7.

Although probiotics are defined as “live microorganisms that, when administered in adequate amounts, confer a health benefit on the host” by the Food and Agriculture Organization of the United Nations and the World Health Organization [1], growing evidence has shown that not only living bacteria but also dead bacteria can exert probiotic effects [40,41]. In addition, Hayashi et al. showed that heat-killed CBM 588 induced IL-10 production by macrophages [6]. We investigated whether UV-killed CBM 588 prolonged the lifespan of *C. elegans*. Notably, the lifespan of worms fed UV-killed CBM 588 was increased compared with UV-killed OP50 (Figure 2). Therefore, not only living CBM 588 but also dead CBM 588 exerted longevity effects in *C. elegans*.

### 3.3. CBM588 Improved Locomotion during Aging in C. elegans

**Figure 3:**
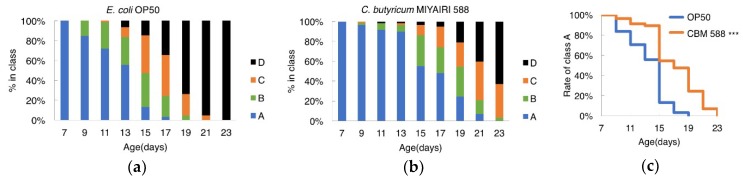
CBM588 improved locomotion during aging in *C. elegans*. (**a**,**b**) Locomotory activity of *C. elegans* fed OP (**a**) or CBM 588 (**b**). Animals were grouped into the following four classes based on their locomotion: class A, robust, coordinated sinusoidal locomotion (blue bars); class B, uncoordinated and/or sluggish movement (green bars); class C, no forward or backward movement, but head movements or shuddering in response to prodding (orange bars); and class D, dead animals (black bars). The frequency of each class at the indicated time point is indicated. *n* ≥ 57 for each assay. (**c**) The difference in the frequency of “Class A” worms was analyzed using the log-rank test. *** *p* < 0.001. OP50, *n* = 61, CBM 588, *n* = 57.

To determine whether CBM 588 improved the decline of locomotion during aging, the motility of worms was scored. The scores of animals fed CBM 588 were higher than those of animals fed OP50 at every age tested (Figure 3a,b). The percentage of worms in class “A,” wherein animals exhibited spontaneous movement or vigorous locomotion in response to prodding, was significantly increased in the CBM 588-fed group (Figure 3c). These results support the hypothesis that CBM 588 not only extends the lifespan of *C. elegans* but also improves health.

### 3.4. Genes That Were Regulated by the CBM 588 Feeding

**Figure 4:**
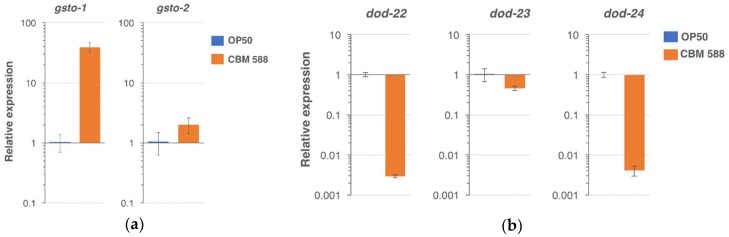
Relative gene expression (determined using real-time PCR) in the animals fed CBM 588 in comparison with control (OP50). (**a**) The *gsto-1* and *gsto-2* expression was upregulated by the CBM 588 feeding. (**b**) The expression of *dod-22*, *dod-23*, *dod-24* was downregulated by CBM 588. Error bars indicate S.D. *n* = 3 biological replicates.

To characterize the genes that were regulated by feeding with CBM 588, RNA sequencing was performed (Tables S1 and S2). A total of 88 genes were shown to be increased in the CBM 588-fed group in comparison with the control-fed group with Log_2_FC ≥ 2 and *p* < 0.05 (Table S3). A total of 459 genes were decreased in the CBM 588-fed group with Log_2_FC ≤ 2 and *p* < 0.05 (Table S4). Significantly enriched Gene Ontology (GO) in the CBM 588-fed group is listed in Table S5 for upregulated genes and in Table S6 for downregulated genes. Within the upregulated genes, the top-ranked GO term for molecular function (MF) was “protein heterodimerization activity” that includes genes encoding for histone components. Because the second-ranked “structural constituent of cuticle” was also significant in the downregulated genes, this GO term was not specifically enriched in the upregulated genes. The expression of genes encoding for glucuronosyltransferases, endopeptidases and heme-binding proteins was also induced. *cpr-5*, an endopeptidase gene upregulated by CBM 588, has been reported to be induced by *Lactobacillus acidophilus* NCFM in a *pmk-1*/p38 MAPK-dependent manner [24]. Notably, “glutathione transferase activity” was significantly enriched in genes that were upregulated by CBM 588. The Omega class glutathione transferase gene *gsto-1* has been reported to play key roles in counteracting environmental stress [42], and *gsto-2* is a paralog of *gsto-1*. Fold-changes of the *gsto-1* and *gsto-2* gene were 45.5 and 5.3, respectively (Table S2). Using real-time PCR, we confirmed that the mRNA expression of *gsto-1* and *gsto-2* was increased in the CBM-fed group compared with the control-fed group (fold-change: *gsto-1*, 39.3 ± 7.1 (S.D.); *gsto-2*, 2.0 ± 1.1) (Figure 4a and Figure S1). Meanwhile, the top-ranked GO term for biological process (BP) in the downregulated genes was “defense response” (Table S6). Intriguingly, dod (downstream of DAF-16) genes such as *dod-22* and *dod-24*, were found in the “defense response” category (Table S6). Tepper et al. has reported class I (upregulated) and class II (downregulated) DAF-16-responsible genes extracted from a meta-analysis, and *dod-23*, *dod-22*, and *dod-24* were the very top 3-ranked genes in class II [43]. The *dod-22*, *dod-23*, and *dod-24* genes were all decreased in the CBM 588-fed group in RNA sequencing (fold-change: 0.029, 0.306, and 0.002, respectively) (Table S2), and also in real-time PCR (0.0029 ± 0.0002 (S.D.), 0.460 ± 0.058, 0.0042 ± 0.0012, respectively) (Figure 4b and Figure S1). In contrast to class II, none of the class I genes listed in Table 1 of the Tepper’s report [43], was found in Table S3. These results suggest that the DAF-16-dependent class II pathway but not class I, may be modulated by feeding with CBM 588.

### 3.5. CBM 588 Increased Resistance to Biological Stresses in C. elegans

**Figure 5:**
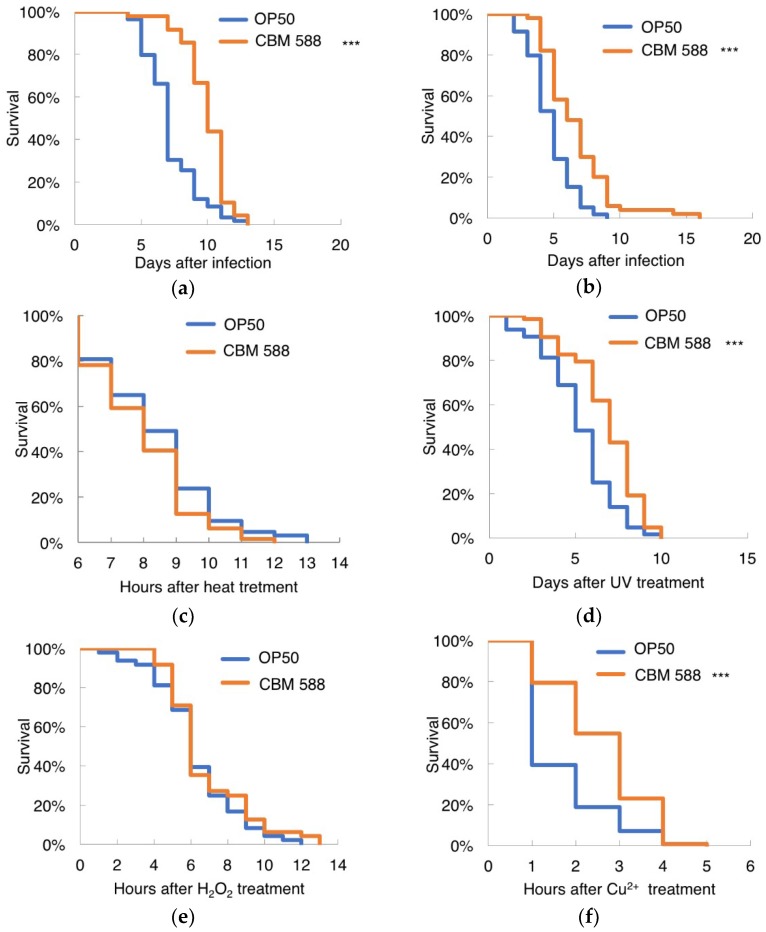
Susceptibility of CBM 588-fed worms to stresses. (**a**,**b**) Survival of *C. elegans* infected with pathogenic bacteria, *Salmonella enterica* (**a**) or *Staphylococcus aureus* (**b**). (**a**) OP50-fed group, *n* = 59; CBM 588-fed group, *n* = 48. (**b**) OP50-fed group, *n* = 59; CBM 588-fed group, *n* = 50. (**c**–**f**) Susceptibility of worms fed CBM 588 to heat stress (**c**), UV irradiation (**d**), hydrogen peroxide (**e**) and Cu^2+^ (**f**). (**c**) OP50, *n* = 64; CBM 588, *n* = 63. (**d**) OP50, *n* = 64; CBM 588, *n* = 63. (**e**) OP50, *n* = 48; CBM 588, *n* = 48. (**f**) OP50, *n* = 63; CBM 588, *n* = 62. Animals were fed OP50 (control) or CBM 588 from an age of 3 days and then exposed to the individual stresses at the ages described in the Materials and methods. Worms were 7 days old (**a**–**c**) or 9 days old (**d**–**f**) on the graph’s nominal Day 0. The survival curves were compared with those of worms fed OP50. *** Statistical significance at *p* < 0.001, log-rank test.

*Salmonella* spp., which are Gram-negative bacteria, and *Staphylococcus aureus*, which are Gram-positive bacteria, shorten the lifespan of worms [23,44,45,46]. We found that probiotic bifidobacteria and lactobacilli could make *C. elegans* resilient to *Salmonella* infection [23]. Afterward, Kim et al. reported that the *L. acidophilus* strain NCFM specifically enhances Gram-positive immune responses; conditioning with *L. acidophilus* NCFM significantly prolonged the survival of nematodes exposed to the Gram-positive pathogens *Enterococcus faecalis* and *Staphylococcus aureus*, but this strain had a minimal effect on Gram-negative infection with *Pseudomonas aeruginosa* or *Salmonella enterica* [24]. To investigate the effect of CBM 588 on the biological stresses caused by pathogenic bacterial infection, 7-day-old worms were exposed to *Salmonella enterica* or *Staphylococcus aureus*. Nematodes fed CBM 588 lived longer after being infected with *Salmonella enterica* or *Staphylococcus aureus* compared with worms fed OP50 (Figure 5a,b). In contrast to the results obtained with NCFM, worms fed CBM 588 exhibited increased resistance to both Gram-positive and -negative bacteria, *Staphylococcus aureus* and *Salmonella enterica*.

### 3.6. CBM 588 Increased Resistance to UV Irradiation and Cu^2+^ in C. elegans

We next examined whether CBM 588 increased resistance to physical and chemical stressors. CBM 588 did not prolong the survival time of worms subjected to heat stress (Figure 5c) but did increase the survival time of worms subjected to UV irradiation (Figure 5d). When the animals were exposed to hydrogen peroxide, which is a known source of active oxygen, the survival rates of OP-fed and CBM 588-fed worms were not significantly different (Figure 5e). In contrast, the CBM 588-fed animals lived longer compared to the OP50-fed animals in the presence of Cu^2+^ (Figure 5f). Transgenic animals overexpressing GSTO-1 were hyperresistant to oxidative stress, and silencing the expression of GSTO-1 by RNAi resulted in worms with an increased sensitivity to oxidants and heat shock [42]. Because CBM 588 conferred resistance to UV irradiation and Cu^2+^ but not to heat or H_2_O_2_ oxidative stress, upregulation of GSTO-1 is unlikely to be the underlying mechanism through which CBM 588 increases stress resistance.

### 3.7. CBM 588 Could Extend the Lifespan of Worms through Regulation of the IIS Pathway and the Nrf2 Transcription Factor

**Figure 6:**
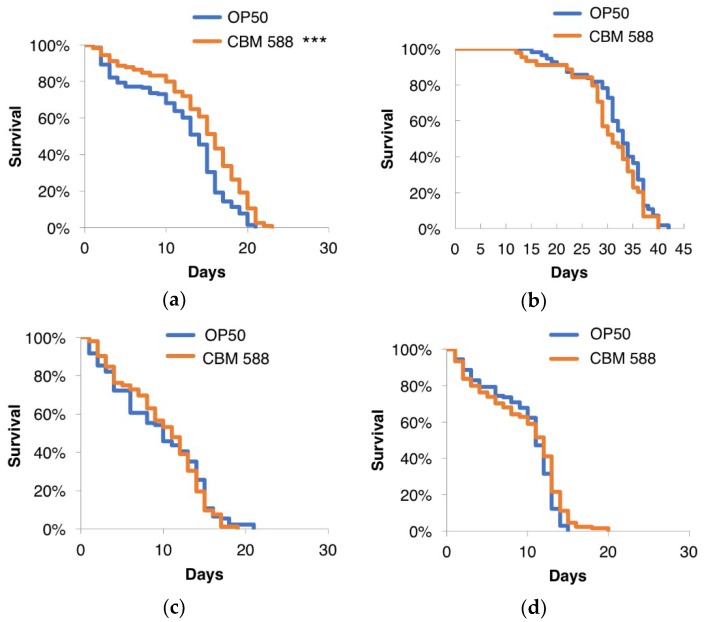
Effects of CBM 588 on the lifespan of *C. elegans* loss-of-function mutants. Survival curves of the wild-type animals (**a**) and the *daf-2* (**b**), *daf-16* (**c**), and *skn-1* mutants (**d**). CBM 588 failed to extend the lifespan of these mutants. The survival curves were compared with those of worms fed OP50. *** Statistical significance at *p* < 0.001, log-rank test. Detailed lifespan data and statistics are provided in Table S7.

The insulin/IGF-1-like signaling (IIS) pathway plays a central role in stress resistance and longevity. In *C. elegans*, IIS is initiated by the binding of insulin-like peptides to the receptor DAF-2, which eventually leads to the activation of downstream IIS kinases, which phosphorylate and inhibit the FOXO transcription factor DAF-16 [47,48,49,50]. Moreover, transcriptional profiling using RNA sequencing implicated the involvement of the DAF-16-dependent class II pathway. To investigate whether the IIS pathway is involved in the prolongevity effects of CBM 588, the lifespans of the *C. elegans* loss-of-function mutants *daf-2* and *daf-16* were measured. CBM 588 failed to prolong the lifespan of strains harboring individual mutations in *daf-2* or *daf-16* (Figure 6). SKN-1 (an ortholog of mammalian Nrf2) is one of the key factors that regulates the stress responses and longevity of *C. elegans* [51,52]. Notably, CBM 588 failed to extend the lifespan of the *skn-1* mutants (Figure 6). These results suggest that both DAF-16 and SKN-1 may be required for the CBM 588-mediated lifespan extension. Because SKN-1 has been shown to be directly suppressed by DAF-2 [53], DAF-2 signaling via SKN-1 activity may also regulate the lifespan of worms fed CBM 588. The p38 MAPK pathway activates SKN-1 in several contexts, including pathogen infection and stress responses [54,55,56], raising the possibility that SKN-1 activation via the p38 MAPK pathway contributes to the prolongevity of animals fed CBM 588. However, we could not determine whether *pmk-1*/p38 MAPK is required for the prolonged lifespan caused by CBM 588 because the *pmk-1* mutation caused severe egg-laying defects when these mutants were fed CBM 588 (data not shown). Further analyses will be necessary to elucidate the role of the p38 MAPK pathway in the increased lifespan resulting from CBM 588. The long-lived mutants *daf-2* and *age-1* exhibited increased resistance to heat [57], oxidants [58,59], UV [60], and metal stress [61], indicating that longevity and stress resistance are closely related. Based on our results from the CBM 588-mediated lifespan extension, suppression of DAF-2 may partially mediate the increased resistance of animals fed CBM 588. However, the possibility that the impact of CBM 588 on longevity was insufficient to further extend the lifespan of the long-lived *daf-2* mutants, cannot be ruled out. In addition, the experiments using mutants that were not outcrossed with our own N2 strain, require caution in the interpretation of these results, because it has been reported that the impact of simply a different N2 strain on lifespan can be huge [62].

## 4. Conclusions

In the present study, we demonstrated for the first time that CBM 588 extends the lifespan of *C. elegans*. The involved mechanisms seemed to depend on the DAF-16/FOXO and the SKN-1/Nrf2 transcription factors. Our transcriptional profiling comparing CBM 588-fed and control-fed animals suggested that DAF-16-dependent class II genes were regulated by CBM 588. Taken together with the fact that reduced IIS extends the lifespan of *C. elegans* by upregulating stress response (class I) and downregulating other (class II) genes in a DAF-16-dependent manner [43], CBM 588 may exert its prolongevity through modulating of the DAF-16 class II pathway. Because DAF-16/FOXO is well-conserved transcription factor that regulate longevity among animal species including humans, it would be worth studying the DAF-16-dependent mechanisms that govern the effects of the probiotic CBM 588.

## Supplementary Materials

The following are available online at http://www.mdpi.com/2072-6643/10/12/1921/s1, Figure S1: Gene expression (real-time PCR) in the animals fed OP50 or CBM 588. Table S1: Mapping statistics. Table S2: Differential expression analysis using DESeq (CBM 588 vs. OP50 (control)). Table S3: Genes upregulated in CBM-fed animals. Table S4: Genes downregulated in CBM-fed animals. Table S5: Gene ontology (GO) enrichment for genes upregulated by CBM 588. Table S6: Gene ontology (GO) enrichment for genes downregulated by CBM 588. Table S7: Summary of lifespan experiments.
