# Supplementary material for: Clostridium butyricum MIYAIRI 588 Increases the Lifespan and Multiple-Stress Resistance of Caenorhabditis elegans
Source: Nutrients. 2018 Dec 5;10(12):1921. doi: 10.3390/nu10121921 (PMC6316807; doi:10.3390/nu10121921)
Supplement: Supplementary file 1 [file nutrients-10-01921-s001.zip › TableS7.docx]

Table S7. Summary of lifespan experiments

| Strain/Food | Mean lifespan  (days) | Maximum lifespan  (days) | Number of animals  (n) | *P*-Value |
| --- | --- | --- | --- | --- |
| WT/OP50 | 17.6±0.4 | 22.7±0.2 | 122 |  |
| WT/CBM588 | 22.3±0.4 | 27.4±0.3 | 109 | <10^-3^ |
| WT/Mixture | 19.3±0.4 | 25.0±0.3 | 112 | <10^-3^ |
| WT/OP50 | 18.4±0.6 | 23.9±0.5 | 56 |  |
| WT/CBM588 | 20.7±0.5 | 26.2±0.4 | 72 | <10^-3^ |
| WT/UV-killed OP50 | 19.1±0.6 | 25.2±0.3 | 57 |  |
| WT/UV-killed CBM588 | 21.9±0.4 | 26.3±0.8 | 55 | 2.0x10^-2^ |
| WT/OP50 | 15.4±0.5 | 22.8±0.2 | 141 |  |
| WT/CBM588 | 17.9±0.5 | 24.4±0.2 | 125 | <10^-3^ |
| *skn-1*/OP50 | 12.9±0.6 | 20.4±0.6 | 94 |  |
| *skn-1*/CBM588 | 13.5±0.5 | 19.8±0.3 | 92 | 0.74 |
| *daf-2*/OP50 | 32.5±0.9 | 39.9±0.6 | 55 |  |
| *daf-2*/CBM588 | 30.9±1.1 | 38.8±0.6 | 44 | 0.28 |
| *daf-16*/OP50 | 13.1±0.4 | 17.5±0.1 | 140 |  |
| *daf-16*/CBM588 | 13.2±0.4 | 19.1±0.4 | 134 | 0.10 |
