# Supplementary figures and images for: Clostridium butyricum MIYAIRI 588 Increases the Lifespan and Multiple-Stress Resistance of Caenorhabditis elegans
Source: Nutrients. 2018 Dec 5;10(12):1921. doi: 10.3390/nu10121921 (PMC6316807; doi:10.3390/nu10121921)

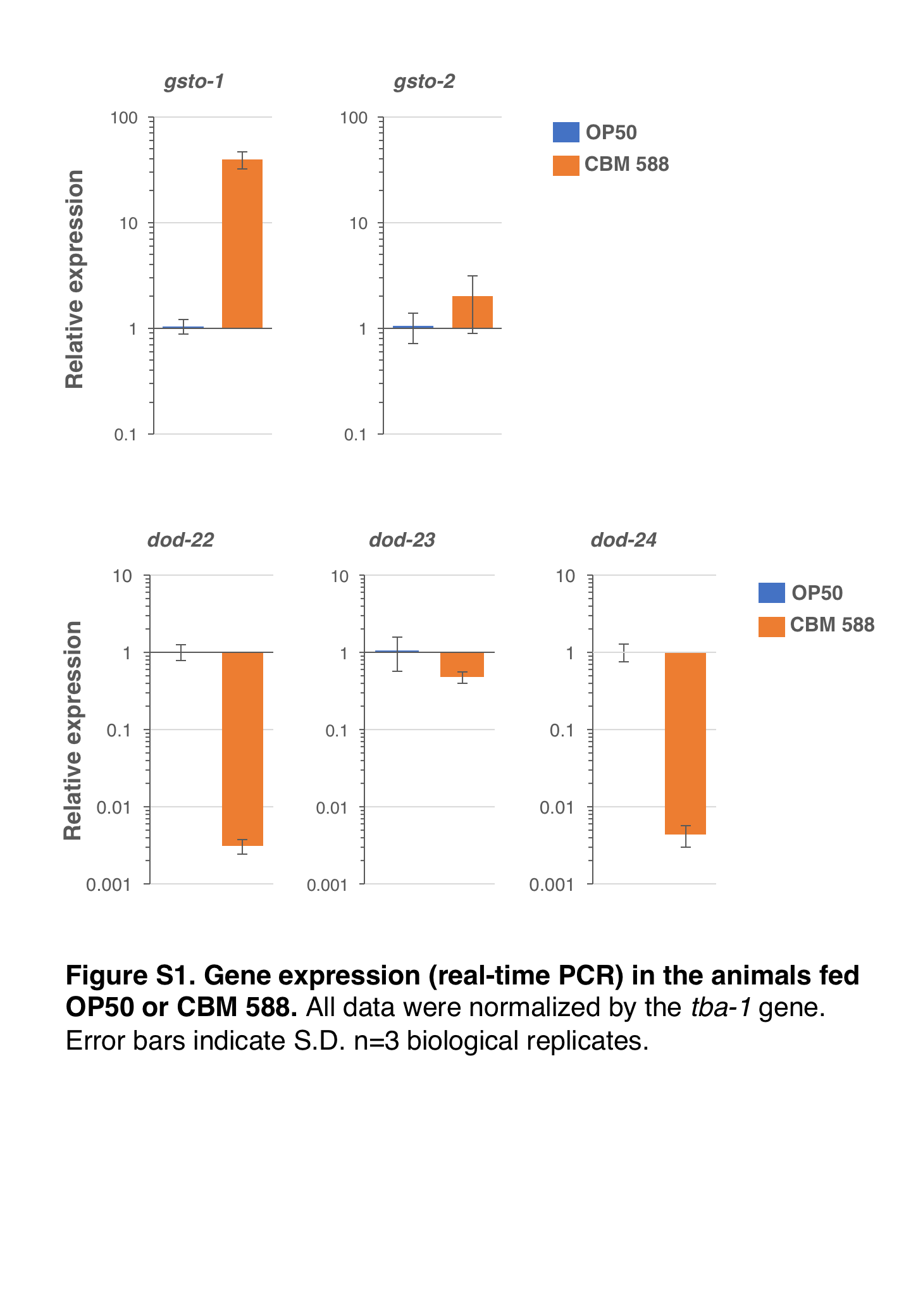

Supplement: Supplementary file 1 [file nutrients-10-01921-s001.zip › Figure S1.tif]
